# Microhomology-Mediated Nonhomologous End Joining Caused Rearrangement of *EMD* and *FLNA* in Emery-Dreifuss Muscular Dystrophy

**DOI:** 10.3389/fgene.2021.786294

**Published:** 2021-12-17

**Authors:** Danyu Song, Xiaomei Li, Wei Wei, Xueqin Liu, Lin Wu, Hui Xiong

**Affiliations:** ^1^ Department of Pediatrics, Peking University First Hospital, Beijing, China; ^2^ Department of Pediatric Cardiology, The First Hospital of Tsinghua University (Beijing Huaxin Hospital), Beijing, China; ^3^ Beijing Kangso Medical Inspection Co., Ltd., Beijing, China; ^4^ Department of Cardiology, Peking University First Hospital, Beijing, China

**Keywords:** Emery–Dreifuss muscular dystrophy, microhomology-mediated nonhomologous end joining, EMD, FLNA, right heart abnormalities

## Abstract

**Background:** Emery–Dreifuss muscular dystrophy (EDMD) is a rare disease characterized by early joint contractures, slowly progressive muscular dystrophy, and cardiac involvement, which includes arrhythmia, dilated cardiomyopathy, hypertrophic cardiomyopathy, heart failure, and sudden death.

**Methods:** Clinical data of the proband and family members were collected. The next-generation sequencing technology was used to analyze the pathogenic variants and copy number variations. Polymerase chain reaction was used to sequence the breakpoints of gene locus rearrangements.

**Results:** Here, we report two siblings with EDMD in a family. The proband, a 17-year-old boy, manifested a dilated right heart, bradycardia, mild muscle weakness, and joint contractures. His younger brother only showed a mild bowing limitation with elevated creatine kinase. Next-generation sequencing revealed the complete deletion of *EMD* and a rearrangement in *FLNA* (exon29_48dup) in these two patients. The *EMD* deletion and partial *FLNA* duplication were accompanied by a 5 bp overlap (GTCCC) on the background of the *FLNA-EMD* inversion. These findings support the pathogenic mechanism of microhomology-mediated nonhomologous end joining.

**Conclusion:** We report two siblings with complete *EMD* deletion and *FLNA* duplication in a family. A microhomology-mediated nonhomologous end joining event involving *EMD* and *FLNA* acts as the underlying mechanism.

## Introduction

Emery–Dreifuss muscular dystrophy (EDMD) is a rare inherited disease that is characterized by early joint contractures, slow-progressive muscular dystrophy, and cardiac involvement (Madej-Pilarczyk, 2018; Heller et al., 2020), including arrhythmias, cardiomyopathy, heart failure, and sudden death. In the Online Mendelian Inheritance in the Man (OMIM) database, EDMD is divided into seven phenotypes, namely, EDMD 1 to 7, based on their causative genes and inherited manner. These genes include *EMD*, *LMNA*, *SYNE1*, *SYNE2*, *FHL1*, and *TMEM43* (Heller et al., 2020). In addition, *SUN1*, *SUN2*, and *TTN* can also cause EDMD (Heller et al., 2020). Among these causative genes, *EMD* and *LMNA* are the most common (Bonne and Quijano-Roy, 2013). EMD, located in Xq28, is assigned to the X-linked EDMD (also named EDMD 1). In patients with X-linked EDMD, joint contractures (elbows, Achilles tendons, and cervical spine) frequently emerge in the first decade of life, and muscle weakness (in a “humeroperoneal” pattern) usually occurs in the second decade of life. Cardiac involvement typically starts after joint contractures and muscle weakness. Here, we report two EDMD patients with an *EMD* deletion and partial *FLNA* (a neighboring gene of *EMD*) duplication in an unconsanguineous family, supporting the pathogenic mechanism of microhomology-mediated nonhomologous end joining.

## Materials and Methods

### Clinical Data Collection

The research protocol (2015[916]) was reviewed and approved by the Ethics Committee of Peking University First Hospital. Written informed consent, which also included consent for the publication of medical data, was obtained from the patients and their parents.

Medical records of the patients were reviewed. Clinical and laboratory data, including age of onset, onset of recognizable symptoms, motor development, contractures, creatine kinase (CK) levels, electrocardiography (ECG), Holter, ultrasound cardiography (UCG), cardiac magnetic resonance imaging (MRI), limb muscle MRI, and muscle biopsy findings, were collected.

### Variant Screening and Pathogenicity Verification

Genomic DNA of the proband, the younger brother, and their parents was extracted from leukocytes isolated from peripheral blood. A muscular disease gene panel and whole exome sequencing (WES) were successively used to detect genetic variations in the proband. Candidate variants in the probands were validated by Sanger sequencing in the family. Disease association databases and population databases, including the HGMD (HGMD: http://www.hgmd.cf.ac.uk/ac/index.php), LOVD (LOVD: http://www.dmd.nl/), ClinVar database (https://www.ncbi.nlm.nih.gov/clinvar/), 1000 Genomes (http://www.1000genomes.org/), ExAC (ExAC: http://exac.broadinstitute.org/), and gnomAD (gnomAD: http://gnomad.broadinstitute.org/) databases, were used to identify previously reported mutations and discover potential novel mutations. Predictions for the pathogenicity of missense mutations were obtained using PolyPhen-2, SIFT, and Mutation Taster. The predicted splicing mutations were tested through the Human Splicing Finder. The pathogenicity of candidate variants was classified according to the American College of Medical Genetics and Genomics and the Association for Molecular Pathology (ACMG-AMP) guidelines (Richards et al., 2015).

### Next-Generation Sequencing Data Analysis for Copy Number Variation

Next-generation sequencing (NGS) data were also analyzed to detect copy number variations (CNVs) by comparing the number of sequence reads between the patient and control samples. First, the number of reads in each amplicon was counted for the patient and control samples. For the purposes of this comparison, sequencing data from at least three control samples (genomic sequencing had been performed for patients diagnosed with other diseases) were used as the reference. Subsequently, the patient/control ratios were calculated by dividing the number of patient reads in each amplicon with the average of the total reads regarding this amplicon from control samples. For the genes, including *EMD* and its neighboring gene *FLNA*, located on the X chromosome, patient/control ratios near 0.5 indicated the presence of heterozygous deletions in female patients (control samples were female). Ratios near 0 indicated deletions in male patients (control samples were male). In contrast, ratios near 1.5 indicated the presence of heterozygous duplications in female patients (control samples were female), and ratios higher than 1.75 indicated duplications in male patients (control samples were male). Polymerase chain reaction (PCR) was used to confirm the deletion of *EMD* in male patients. Statistical results were illustrated using GraphPad Prism 8 software (GraphPad Software, La Jolla, CA).

### PCR for Deletion Validation and the Sequencing of Breakpoints of the CNVs

PCR and Sanger sequencing were performed to verify *EMD* deletion and to sequence the breakpoints of the CNVs. Primers were designed separately for exons 1–6 of *EMD* using Primer 5.0 (Premier Biosoft, Palo Alto, CA, United States). The two primers designed for exon 1 were Primer F 5′-ACC​GCG​AGA​CCT​TTT​GCT​C-3′ and Primer R 3′-GCG​AGG​CTC​TCA​CCA​GAG​AA-5′. The two primers designed for exon 2 were Primer F 5′-CGC​ACG​GGC​CTG​TAG​TAG​GT-3′ and Primer R 3′-GAC​GAA​GTC​GGG​TGA​AGG​TG-5′. The two primers designed for exon 3 were Primer F 5′-CGG​CCA​GGA​TCA​ACT​CGT​AG-3′ and Primer R 3′-TCC​TAA​GGC​TGC​TGG​AGT​GG-5′. The two primers designed for exon 4 were Primer F 5′-ACC​TTC​ACC​CGA​CTT​CGT​CA-3′ and Primer R 3′-TCC​TGA​ATG​GCT​CTG​GGT​GT-5′. The two primers designed for exon 5 were Primer F 5′-GAG​CCA​TTC​AGG​AGG​GTG​TG-3′ and Primer R 3′-GCT​GAA​ACA​GGG​CGG​TAG​TG-5′. The two primers designed for exon 6 were Primer F 5′-TGT​GGG​TTC​CTG​GCC​TCT​AA-3′ and Primer R 3′- AAA​AAT​GCG​GAC​CCA​ACA​AA-5′. For the breakpoints, two primers were designed for a location in exon 2 of *FLNA* (Primer F GRCh37/hg19, chrx:153599438-1535994, 5′-CAC​TTC​AGG​TGC​TCG​TT-3′) and exon 29 of *FLNA* (Primer R GRCh37/hg19, chrx:153585865-153585883, 3′-GGA​TCT​CGT​CAC​CAC​CGT​A-5′). PCR amplifications were performed using GoldStar Taq DNA Polymerase in an Applied Biosystems Veriti Thermal Cycler (4375305, Thermo Fisher Scientific, Waltham, MA, United States). Each PCR amplification was repeated for 35 cycles (30 s at 95°C, 30 s at 60°C, and 45 s at 72°C). Before sequencing, the PCR products were analyzed by electrophoresis on a 2% agarose gel.

## Results

### Clinical Findings of the Proband

**FIGURE 1:**
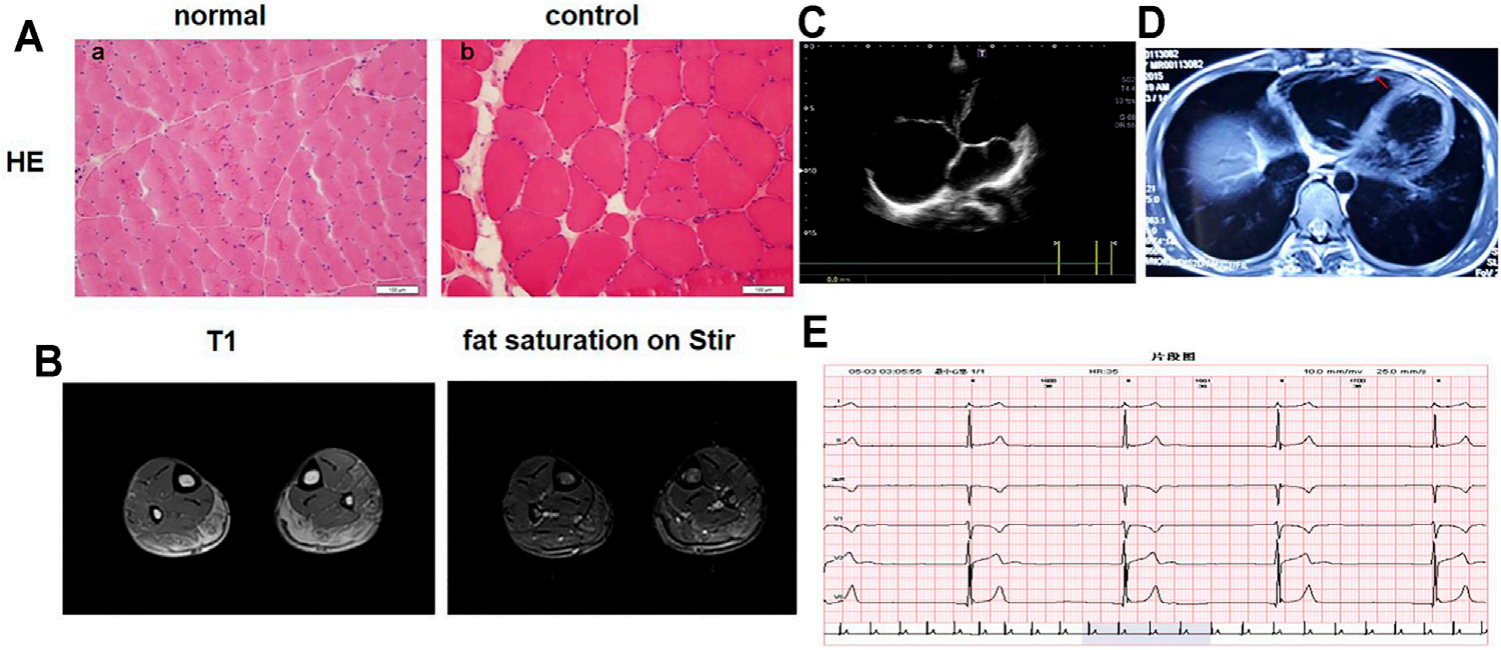
Clinical Results. **(A)** Muscle biopsy. (a) Hematoxylin and eosin (H&E) staining of serial sections of the skeletal muscle from a normal control. (b) H&E staining of the skeletal muscle of the proband shows dystrophic changes: variability in the size of muscle fibers, fibrosis, and fat replacement. **(B)** Limb muscle MRI showed infiltration of fatty tissues in the soleus. **(C)** Echocardiography showed enlargement of the right heart. **(D)** Cardiac magnetic resonance imaging revealed an obviously enlarged right heart and evident thinning of the lateral wall of the right ventricle. The red arrows show fatty tissues in the local cardiac muscle. **(E)** Holter monitoring showed bradycardia and a junctional ectopic rhythm.

The proband was referred to our hospital at the age of 17 due to suspected “skeletal muscle cardiomyopathy”. He started to feel physical activity-related tiredness and chest distress at the age of 13. He was suspected of having “myocarditis” and was given nutritional therapy without regular follow up. At the age of 15, he visited the local hospital again due to poor exercise tolerance and chest discomfort. Subsequently, bradycardia and enlargement of the right heart were found by Holter monitoring and UCG. Due to the symptomatic right heart problem, he was suspected of having “right ventricular cardiomyopathy” and received indolapril as a treatment. At the age of 16, he presented with bilateral brachial muscle pain and aggravated chest distress. His motor and cognitive development was normal. None of his family members exhibited any clinical abnormalies. Physical examination at the age of 17 revealed a heart rate of 56 bpm, normal oxygen saturation, and no dyspnea at rest. Neurological examination showed obvious bilateral brachial and peroneal muscle atrophy, elbow contracture, a mild rigid spine, and a tight Achilles tendon with normal gait. The muscle strength grade of the upper proximal arms was IV^+^, while that of the distal arms was V. The muscle strength grade of the proximal lower limbs was V^−,^ with a distal dorsal flexion power of IV and a plantar flexion power of V^−^. Bilateral knee tendon reflex could not be induced. To confirm the diagnosis, laboratory tests were performed. The level of CK was 500–2,500 U/L (normal 0–200 IU/L). Skeletal muscle biopsy indicated muscular dystrophic changes (Figure 1A). Limb muscle MRI showed infiltration of fatty tissues, predominantly in the soleus (Figure 1B). UCG showed enlargement of the right heart with a left ventricular ejection fraction (LVEF) of 68% (Figure 1C), and the Holter monitoring showed a junctional ectopic rhythm of 36 bpm (Figure 1E). A cardiac MRI subsequently revealed a markedly enlarged right heart, evident thinning of the lateral wall of the right ventricle, and fatty tissues in the local cardiac muscle with LVEF of 57% (Figure 1D). Combining the predominant cardiac involvement, muscle weakness, and joint contractures, he was clinically diagnosed with EDMD. This was confirmed by genetic tests (listed below). Considering bradycardia and the risk of sudden cardiac death, he was referred to implant a cardiac pacemaker. In addition, he was suggested to undergo rehabilitation training with regular follow up.

### Genetic Results

**FIGURE 2:**
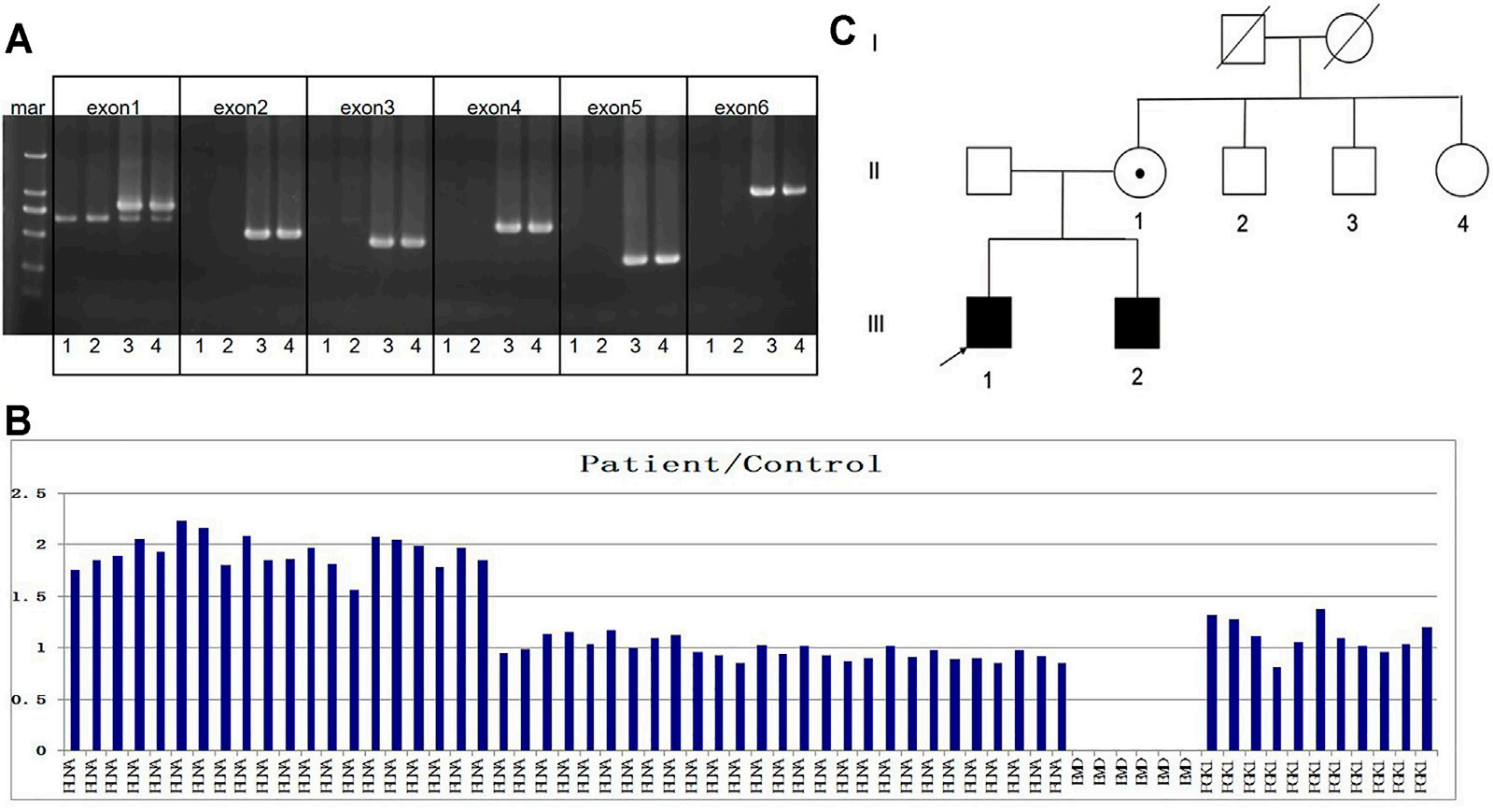
Family pedigree and genetic results. **(A)** In the gel, numbers 1 to 4 represent the proband, his brother, his mother, and the control, respectively. The target bands of *EMD* exons 1–6 were deleted in the proband and his brother. **(B)** Column chart shows ratios between the patient and the control patients in terms of the number of reads, suggesting the deletion of exons 1–6 of *EMD* and the duplication of exons 29–48 of *FLNA* in the proband. **(C)** Family pedigree is shown.

**FIGURE 3:**
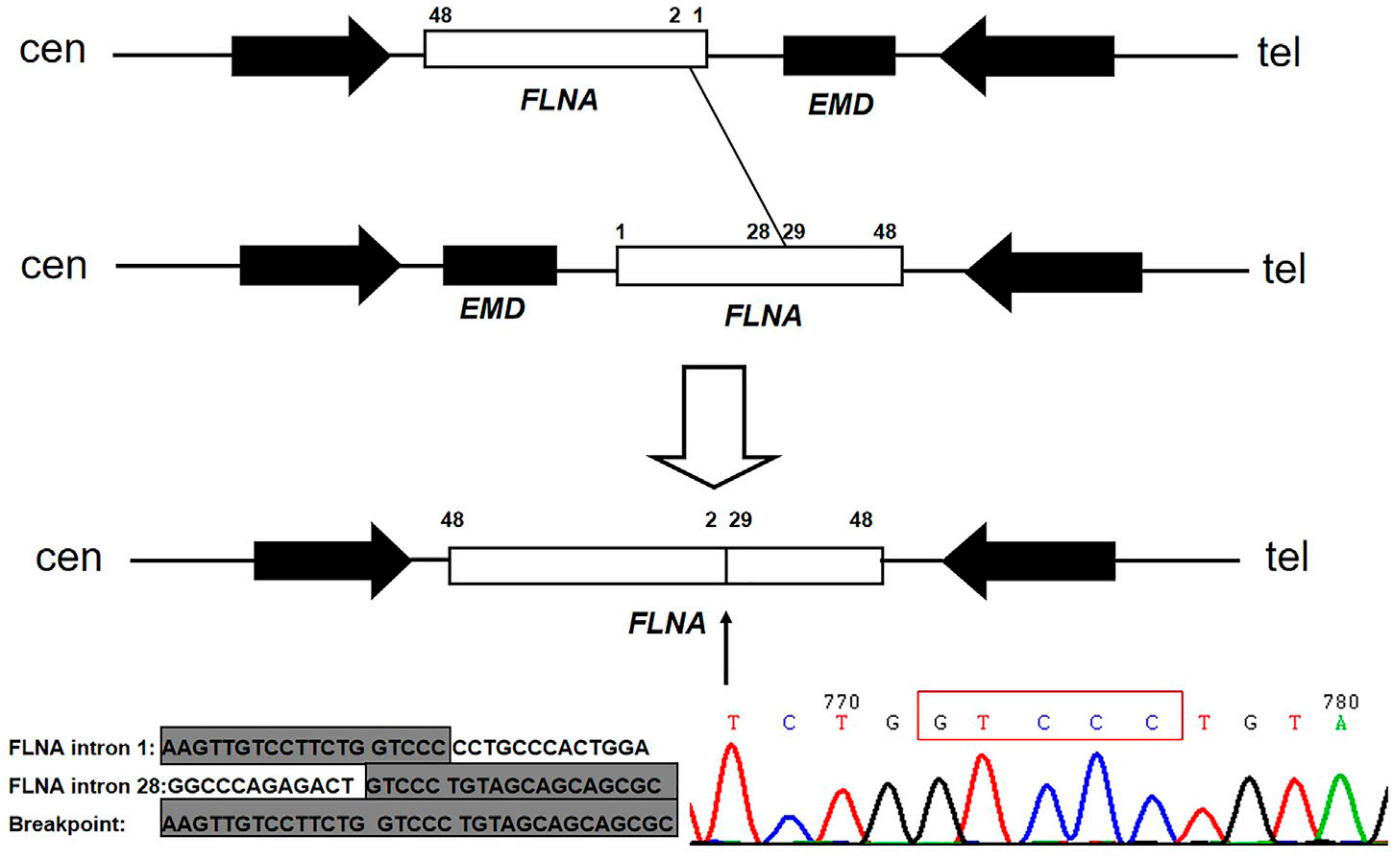
Model of *FLNA* rearrangement and *EMD* deletion. The first allele shows the normal *FLNA-EMD* region. The second allele is a polymorphic inversion containing the entire *FLNA-EMD* region. The unshaded rectangle represents *FLNA*, whereas the shaded rectangle represents *EMD*. Two arrows point to the two large reverted repeats flanking the *FLNA*-*EMD* region. Between homologous X or sister chromatins, 5 bp (GTCCC) overlap between *FLNA* intron 28 and intron 1 on the background of *FLNA*-*EMD* inversion was confirmed.

A muscular disease gene panel that was performed in a local hospital detected no pathogenic variation in the proband. We reanalyzed the NGS data. We did not find any pathogenic variation but found that the depth of coverage was low in the whole region of *EMD.* This suggests *EMD* deletion. The PCR results confirmed the entire deletion of *EMD* (exons 1–6) (Figure 2A) in the proband. Then, we needed to further detect the deleted region and breakpoints. Due to the lack of designed probes and the small size of *EMD*, we failed to analyze the deleted region by using multiplex ligation-dependent probe amplification (MLPA) and array CGH. Finally, we used the NGS data of WES combined with PCR to help verify the region of deletion. The depth of coverage was used to detect the CNV by comparing its copy number ratio between the patient and the control. The ratio was 0 in the region of *EMD*, suggesting the deletion of the entire *EMD* gene (Figure 2B). On the other hand, the ratios were almost all greater than 1.75 in one segment (exon 29_48) and approximately 1 in another segment (exon 2_28) of *FLNA*, a gene adjacent to *EMD*. These findings suggest partial duplication of *FLNA* corresponding to exons 29–48 (Hg19, NM_001110556, Figure 2B). However, due to the lack of reads designed to cover exon 1 of *FLNA*, the WES data were lost in *FLNA* exon 1, which is a non-coding region (Patrosso et al., 1994). The whole *EMD* deletion involving partial *FLNA* duplication was similar to the genomic arrangement of the first reported EDMD case with *EMD* deletion (Small et al., 1997). Therefore, according to the case reported before (Small et al., 1997), we proposed that the genomic rearrangement in the proband may be caused by nonhomologous end joining on the background of *FLNA*-*EMD* inversion. Considering that the duplicated region of *FLNA* (exon 29_48) starts from exon 29, we proposed that one breakpoint in *FLNA* was located in intron 28 of *FLNA*. Besides, *FLNA* exon 2 was unaffected. Therefore, another breakpoint in *FLNA* may occur in intron 1 of *FLNA* to the linkage region of *FLNA*-*EMD*. A model including the inversion of *FLNA*-*EMD* is shown (Figure 3) to explain the rearrangement of *FLNA*. To verify the breakpoints, PCR and Sanger sequencing were performed. Two primer sets were designed to be located in exon 2 of *FLNA* and exon 29 of *FLNA*. After sequencing, deletion boundaries and sequences were found. Between homologous X or sister chromatins, a microhomology-mediated nonhomologous end joining event was confirmed, accompanied by a 5 bp overlap (GTCCC) between *FLNA* intron 28 and *FLNA* intron 1 on the background of *FLNA*-*EMD* inversion.

### Analysis of Other Family Members

The family pedigree is shown in Figure 2C. The family members were then evaluated. The 14-year-old younger brother complained of a mild bowing limitation. His CK level was 549 U/L. His echocardiograph and electrocardiograph revealed no problems. Their parents showed no symptoms, with normal CK and cardiac test results. After the same genetic test, the younger brother was identified as a patient with *EMD* deletion and partial *FLNA* duplication. He was followed up and given rehabilitation training regularly. The mother was confirmed as a carrier.

## Discussion

The proband had symptomatic right heart disease accompanied by mild muscle weakness. Due to his normal primary motor milestones, his mild elbow joint contracture and rigid spine may not have received enough attention. After a detailed physical examination, he was found to have mild muscle weakness, humeroperoneal muscle atrophy, mild elbow joint contracture, and a rigid spine. Based on his muscle weakness, high CK level, and pathological muscular dystrophic changes, the diagnosis of muscular dystrophy could be confirmed. Combining both cardiac and muscle involvement, skeletal muscle cardiomyopathy was confirmed. Given the severe cardiac involvement, humeroperoneal distributed muscle weakness, and special locations of the contractures, he was suspected to have EDMD. Finally, this diagnosis was confirmed by genetic analysis.

*EMD*, located in Xq28, encodes the nuclear protein emerin. The complete deletion of *EMD* in the proband caused an X-linked EDMD phenotype. The cardiac phenotype often emerges within the end of the second decade along the third decade of life in classical X-linked EDMD (Heller et al., 2020). The fact that the obvious right heart involvement started at 14 years of age is unusual. The involvement of the right side of the heart is also not a classical feature of the cardiological spectrum of X-linked EDMD. Significant right heart abnormalities and an early age of onset were features in the proband, with mild weakness and joint contractures. Regarding the mutation type, most mutations reported in *EMD* are nonsense mutations, missense mutations, frameshift mutations, or splicing mutations that cause a truncated protein. There have been five cases in which the complete deletion of *EMD* was reported (Small et al., 1997; Small and Warren, 1998; S et al., 1999), which was found in the proband.

Filamin-A is encoded by *FLNA*. It promotes orthogonal branching of actin filaments and links actin filaments to membrane glycoproteins with wide tissue expression. *FLNA* mutations can cause several severe syndromes, including periventricular nodular heterotopias, otopalatodigital syndromes, skeletal dysplasia, lung involvement, and cardiovascular abnormalities (Sankararaman et al., 2013; van der Werf et al., 2013; Deng et al., 2020; Meliota et al., 2021). In heterozygous females, the *FLNA*-related phenotype ranges from the absence of overall symptoms to severe manifestations. Male patients typically have a very severe phenotype and almost die prenatally or in the first years of life due to multisystem involvement (Sankararaman et al., 2013; van der Werf et al., 2013; Walsh et al., 2017; Cannaerts et al., 2018). This 17-year-old proband only manifested joint contractures and cardiac and muscle involvement without other systems involved. He had relatively minor symptoms compared with typical *FLNA*-related male patients. In addition, the duplication of exons 29–48 of *FLNA* did not influence the primary *FLNA* exons 2–48 (containing the entire open reading frame). Given this consideration, we predicted that *FLNA* is not a causative gene in this patient, despite the *FLNA* rearrangement.

For the molecular pathogenic mechanism, a microhomology-mediated nonhomologous end joining event involving *EMD* and *FLNA*, with a 5 bp overlap (GTCCC), acts as the underlying mechanism. Microhomology-mediated end joining is an error-prone repair mechanism for DNA double-strand breaks. It needs longer microhomology (more than 2 bp) and is independent of Ku70/Ku80, which is distinct from classic nonhomologous end joining (Kent et al., 2015; Sinha et al., 2016; Wang and Xu, 2017; Seol et al., 2018). The *FLNA-EMD* inversion in Xq28 is a common inversion mediated by inverted repeats (Small et al., 1997). The presence of an inversion of the *FLNA-EMD* region among X chromosomes was frequent (33% in females and 11% in males) (Small et al., 1997). The first reported case of EDMD with *EMD* deletion and a partial duplication of *FLNA* revealed an emerin-associated Alu with 2 bp of overlap between one pair of misaligned and inverted repeats (Small et al., 1997). Two other reported cases revealed 2 bp of overlap in *FLNA* (Small and Warren, 1998). They are all different from the proband reported here.

In conclusion, we reported two cases of EDMD with a complete *EMD* deletion and partial *FLNA* duplication. A microhomology-mediated nonhomologous end joining event involving *EMD* and *FLNA* acts as the underlying mechanism.

## Data Availability

The datasets presented in this study can be found in online repositories. The names of the repository/repositories and accession number(s) can be found below: https://www.ncbi.nlm.nih.gov/genbank/, MH151792.
